# Biologically inspired band-edge laser action from semiconductor with dipole-forbidden band-gap transition

**DOI:** 10.1038/srep08965

**Published:** 2015-03-11

**Authors:** Cih-Su Wang, Chi-Shung Liau, Tzu-Ming Sun, Yu-Chia Chen, Tai-Yuan Lin, Yang-Fang Chen

**Affiliations:** 1 Department of Physics, National Taiwan University, Taipei 106, Taiwan; 2 Institute of Optoelectronic Sciences, National Taiwan Ocean University, Keelung 202, Taiwan

## Abstract

A new approach is proposed to light up band-edge stimulated emission arising from a semiconductor with dipole-forbidden band-gap transition. To illustrate our working principle, here we demonstrate the feasibility on the composite of SnO_2_ nanowires (NWs) and chicken albumen. SnO_2_ NWs, which merely emit visible defect emission, are observed to generate a strong ultraviolet fluorescence centered at 387 nm assisted by chicken albumen at room temperature. In addition, a stunning laser action is further discovered in the albumen/SnO_2_ NWs composite system. The underlying mechanism is interpreted in terms of the fluorescence resonance energy transfer (FRET) from the chicken albumen protein to SnO_2_ NWs. More importantly, the giant oscillator strength of shallow defect states, which is served orders of magnitude larger than that of the free exciton, plays a decisive role. Our approach therefore shows that bio-materials exhibit a great potential in applications for novel light emitters, which may open up a new avenue for the development of bio-inspired optoelectronic devices.

In recent years, there's a growing interest to develop laser-based photonic technologies, which have made a tremendous impact on modern science and medical applications, such as sensing and diagnosis^1^. Semiconductor nanowires (NWs) possess several unique electronic and optical properties due to the cylindrical geometry and two-dimensional confinement of electrons and holes, which make them particularly attractive as potential building blocks for nanoscale optoelectronic devices, including light emitting diodes and lasers^2^. As an active photonic device, lasers are composed of a pumped gain medium placed within an optical resonator. Based on these two elements, semiconductor nanowires not only serve as a gain medium but a cavity as well for laser action. Simultaneously, the large refractive index of semiconductors and wire-like geometry enable strongly scattering process existed in the NWs system, and the constructive interference effect consequently results in optical coherent feedback for the outcome lasing modes. Room-temperature lasing has been readily achieved in ZnO, GaN, GaAs and CdSe nanowires due to their large exciton binding energy^3^,^4^,^5^,^6^,^7^. Fruitful derivatives are also promising in wide fields such as biosensors, transistors, gas sensors and solar cells^8^,^9^,^10^,^11^. Among the functional wide-bandgap semiconductors, only limited progress has been made in the field of ultraviolet tin dioxide (SnO_2_) nanowire lasers. Although SnO_2_ has a wide direct bandgap (3.6 eV) and large exciton-binding energy (130 meV), it is so far commonly believed that SnO_2_ is not a suitable ultraviolet (UV) luminescent material due to the dipole-forbidden nature of its bandgap quantum states^12^. Generally, only a broad visible oxygen defect photoluminescence (PL) is observed in most of the reports, since the even-parity symmetry of the conduction band and valence band states in SnO_2_ prohibits the bandgap radiative transition^12^. Until now, only a little attention has been paid to the research for bringing up the related mechanism of SnO_2_ photoemission in ultraviolet range, the underlying origins still remain as an issue of debates^13^,^14^. More theoretical insights are still in need. However, in order to achieve a better UV performance of SnO_2_ NWs and overcome the difficulties for future coming applications, an improvement for the external conversion efficiency is essential and now paramount.

Chicken egg white (albumen), as a kind of ubiquitous nutritious food in our daily life can serve as a new excellent UV-emitting (~340 nm) protein. Compared with traditional laser dyes (e.g. rhodamine 6G (R6G)), the advantages of chicken albumen consist of the biodegradable, bioresorbable and biocompatible characteristics. More attractive, it is cost-effective and environmentally friendly. Very recently, related research has been paid to the field of modern organic optoelectronics, such as light emitters and field effect transistors (FET)^15^,^16^. As a new generation of efficient emitting biomolecule, chicken albumen is expected to be potential for future green technologies.

In this paper, we demonstrate, for the first time a facile and practical strategy to fabricate a newly designed organic-inorganic hybrids composite based on chicken albumen/SnO_2_ NWs. Quite surprisingly, it is observed that the originally undetectable UV fluorescence (387 nm) in pristine SnO_2_ NWs can be drastically enhanced in the albumen/SnO_2_ NWs composite. More excitingly, via effective optical excitation, the coherent laser action can be further sustained and derived. Our strategy to overcome the UV-light-emitting drawback of SnO_2_ NWs is based on the fluorescence resonance energy transfer (FRET). FRET is a physical phenomenon where excitation energy from an excited donor (chicken albumen) is non-radiatively transferred to a proximal ground-state acceptor (SnO_2_ NWs), and the energy transfer process in a FRET system requires good overlap between optical transition bands of donor and acceptor as well^17^. As reported, FRET has been shown to be sufficiently applicable to a variety of biological systems^18^. Our proposed unprecedented FRET-assisted UV-emitting laser device based on albumen/SnO_2_ NWs composite is not only simple but also paves an excellent alternative way of utilizing chicken albumen protein, which may enable to trigger the further development of bio-inspired optoelectronic devices.

## Results

### Morphology and optical characterization of SnO_2_ NWs and chicken albumen

**Figure 1:**
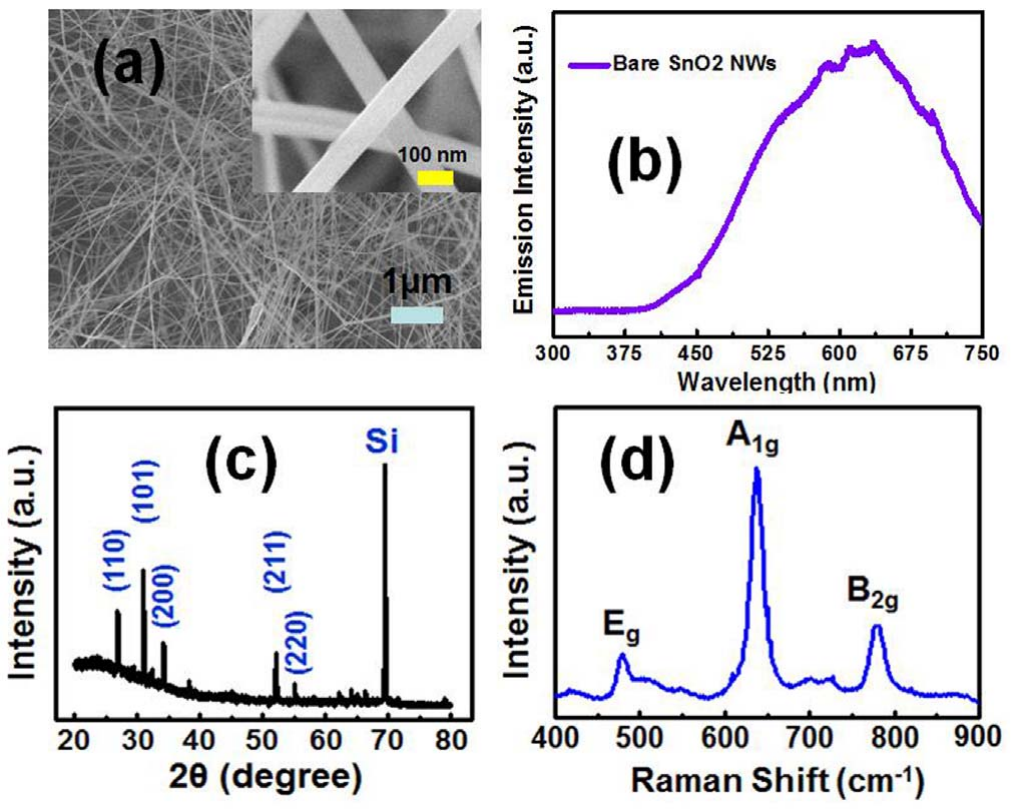
(a) Scanning electron microscope (SEM) image of the as-grown pristine SnO_2_ nanowires (NWs). The inset shows a closer SEM image. (b) Photoluminescence of SnO_2_ NWs. (c) X-ray diffraction pattern of SnO_2_ NWs. (d) Raman scattering spectrum of SnO_2_ NWs.

**Figure 2:**
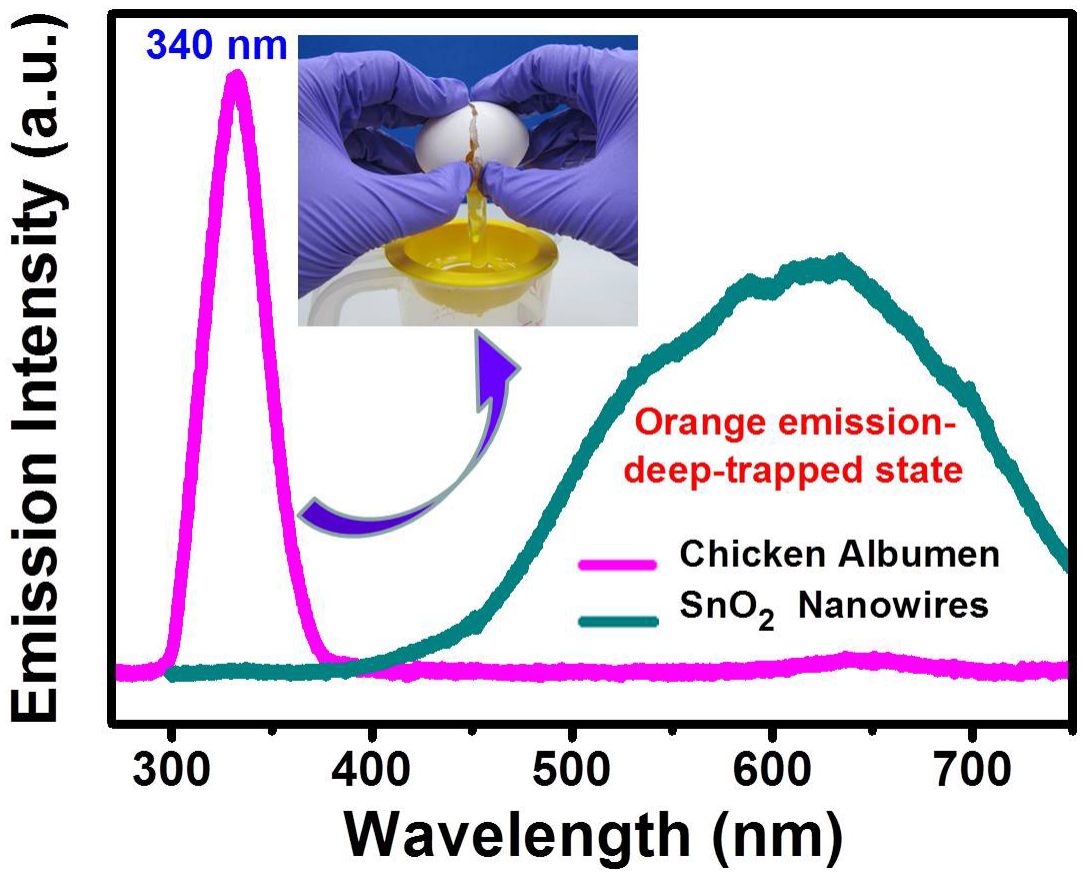
Photoluminescence spectra of chicken albumen (340 nm) and bare SnO_2_ NWs (625 nm). The inset illustrates the separation of egg white (albumen) and egg yolk. (The photograph of the inset was taken by the first author Cih-Su Wang.).

**Figure 3:**
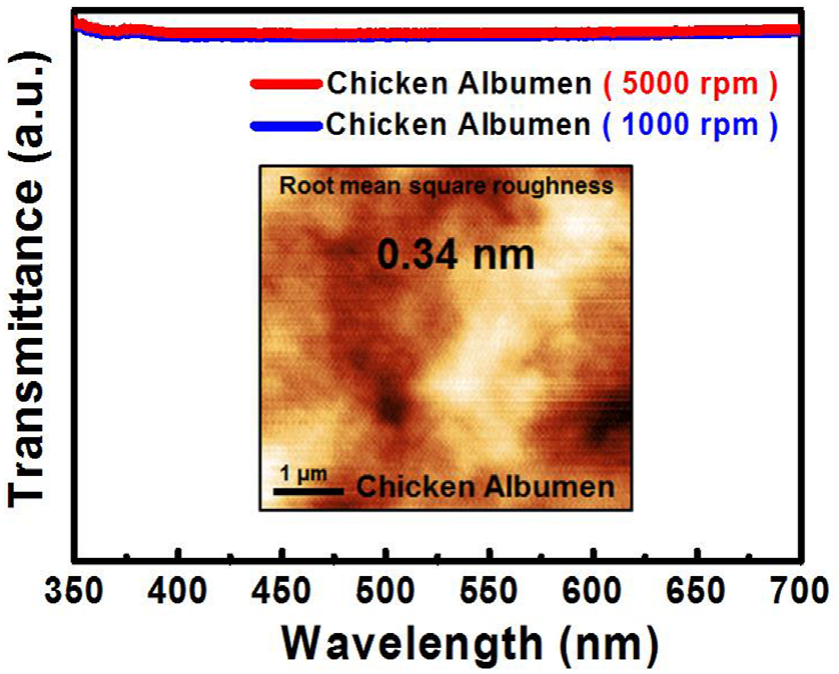
Transmittance spectra of pristine albumen under different spin-coating speed. The inset shows the atomic force microscope image of albumen surface.

Figure 1(a) shows the top view SEM image of the as-synthesized SnO_2_ NWs. It is observed that the nanowires are randomly assembled and closely packed. An inset of Fig. 1(a) reveals a closer view on SnO_2_ NWs. The average length is of about 10 μm, while the diameter is ranging between 70 nm and 150 nm. The XRD pattern of the as-prepared SnO_2_ NWs is shown in Fig. 1(c). It can be seen that all peaks are perfectly indexed to the tetragonal rutile SnO_2_. As additional evidence, a Raman scattering spectrum is shown in Fig. 1(d), in which the three peaks at 475, 630, 768 cm^−1^ correspond to the E_g_, A_1g_, and B_2g_ vibration modes, respectively. All these informations further confirm the existence of the as-grown SnO NWs. For the optical properties, a PL spectrum is first characterized as shown in Fig. 1(b). We can clearly see that only a broad orange emission peak located at 625 nm (2.0 eV) is observed, and no ultraviolet fluorescence can be detected. The detected visible light arising from SnO_2_ is generally believed to stem from the deep-trapped state, which is related to the oxygen vacancies (O_v_) or tin interstitials (Sn_i_)^14^. Figure 2 presents the PL spectra for both of the pristine SnO_2_ NWs and chicken albumen. The UV emission arising from albumen is centered at around 340 nm, and the FWHM is much narrower compared with that of the bare SnO_2_ NWs. It is found that the optical property for albumen is quite stable even under the UV laser pumping. At the beginning, the albumen was spin-coated on a cleaned glass substrate for the PL measurement. The first sample was then stored and preserved in a Petri dish at room temperature. More than our expectation, the PL can still be detected and shown to be stable even after 6 months. Based on these characteristics, albumen from chicken egg reveals one of its advantages as an excellent UV-emitting biomolecule. The inset of Fig. 2 illustrates the separation of egg white (albumen) and egg yolk. In addition to the fluorescence properties, the transmittance spectra of albumen with different spin-coating speed were also shown in Fig. 3. The film thicknesses are about 800 nm (1000 rpm) and 400 nm (5000 rpm), respectively. Both the albumen samples with different thickness show similar transmission properties which indicate good transparency from ultraviolet to visible range. The inset of Fig. 3 shows the topological AFM of the albumen surface, and the root mean square (RMS) roughness is 0.34 nm.

### Photoluminescence and laser action of albumen/SnO_2_ NWs composite

**Figure 4:**
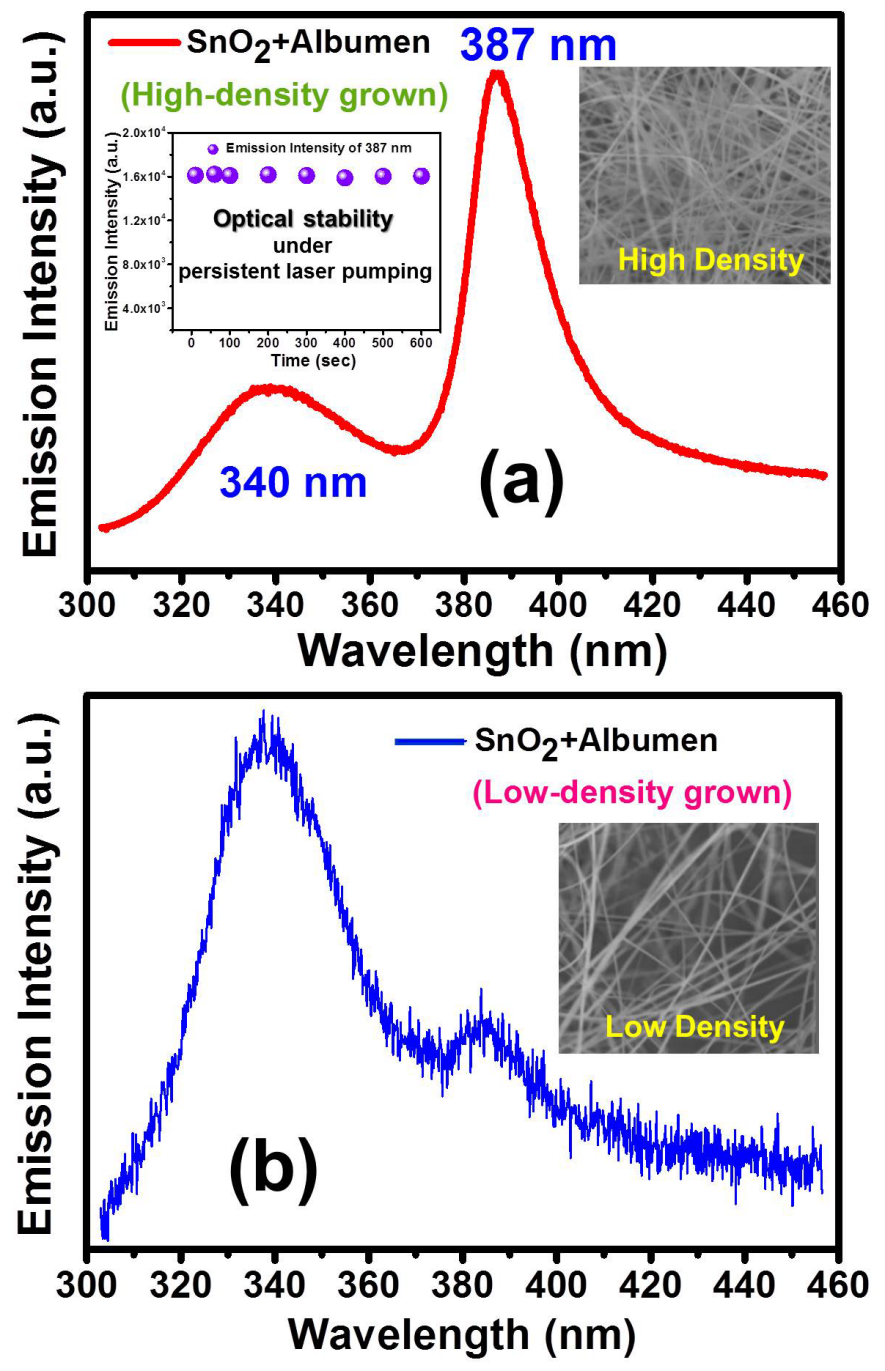
Photoluminescence (PL) spectra of albumen-coated SnO_2_ nanowires (NWs) both for (a) high-density and (b) low density-grown SnO_2_ NWs. The left inset of Fig. 4(a) shows the PL intensity of 387 nm as a function of time. The rest insets show the scanning electron microscope images of SnO_2_ NWs without albumen coating.

Figure 4 shows the PL spectrum of the organic-inorganic hybrid structure of albumen/SnO_2_ NWs composite. At the beginning, no ultraviolet fluorescence can be ever detected from the as-synthesized bare SnO_2_ NWs. However, to our astonishment, when chicken albumen is spin-coated (5000 rpm) onto the SnO_2_ NWs, two UV emission peaks located at the positions of 340 nm and 387 nm emerge. Similar result can both be found in high-density (Fig. 4(a)) and low-density (Fig. 4(b)) grown SnO_2_ NWs coated by albumen. The 340 nm emission is indexed to the existing albumen covered on SnO_2_ NWs, and is basically the same as prior shown in Fig. 2. Compared with the albumen emission, we are more intriguing to characterize the new emerging 387 nm UV fluorescence. Tin dioxide is a direct bandgap semiconductor, however, limited by its dipole-forbidden nature, the bandgap emission with the photon energy of 3.6 eV (~344 nm) is prohibited at room temperature due to the selection rule^14^. In addition to the former described deep-trapped state (~2.0 eV) resulting in the visible light, a shallow-trapped state (3.2 eV) within the bandgap of SnO_2_ has also been found^14^. Via the comparison of Figs. 4(a) and 4(b), it is apparent that the photoemission of 387 nm strongly depends on the density of SnO_2_ NWs, and decreases as the NWs turn to be sparse. Herein, the observed UV fluorescence centered at 387 nm (3.2 eV) is believed due to the shallow state of SnO_2_ NWs as shown in the early reports^4^,^13^,^14^. To test the photo-stability of the emission of 387 nm from the albumen/SnO_2_ composite, the PL intensity was measured at regular time interval under a persistent laser pumping with fixed energy (left inset of Fig. 4(a)). All the recorded data remained in the same order of magnitude with a considerable stability of more than 600 sec. Therefore, an excellent photo-stability of our sample is further confirmed for practical applications. It is worth noting that the visible defect emission of SnO_2_ NWs remains nearly the same before and after albumen coating. Since the visible emission is mainly due to the oxygen vacancies and independent of the albumen coating, the emission spectra are therefore not shown here. The rest right insets of Fig. 4 show the SEM images of dense and sparse SnO_2_ NWs without albumen coating. Concerning that the SnO_2_ NWs are randomly assembled, the coating of albumen might be partially uneven. However, supported by the transmittance spectra in Fig. 2, the similar fine transparency of albumen both for 1000 rpm and 5000 rpm indicates that the thickness effect on the intensity of the photoemission (387 nm) can be fairly ignored.

**Figure 5:**
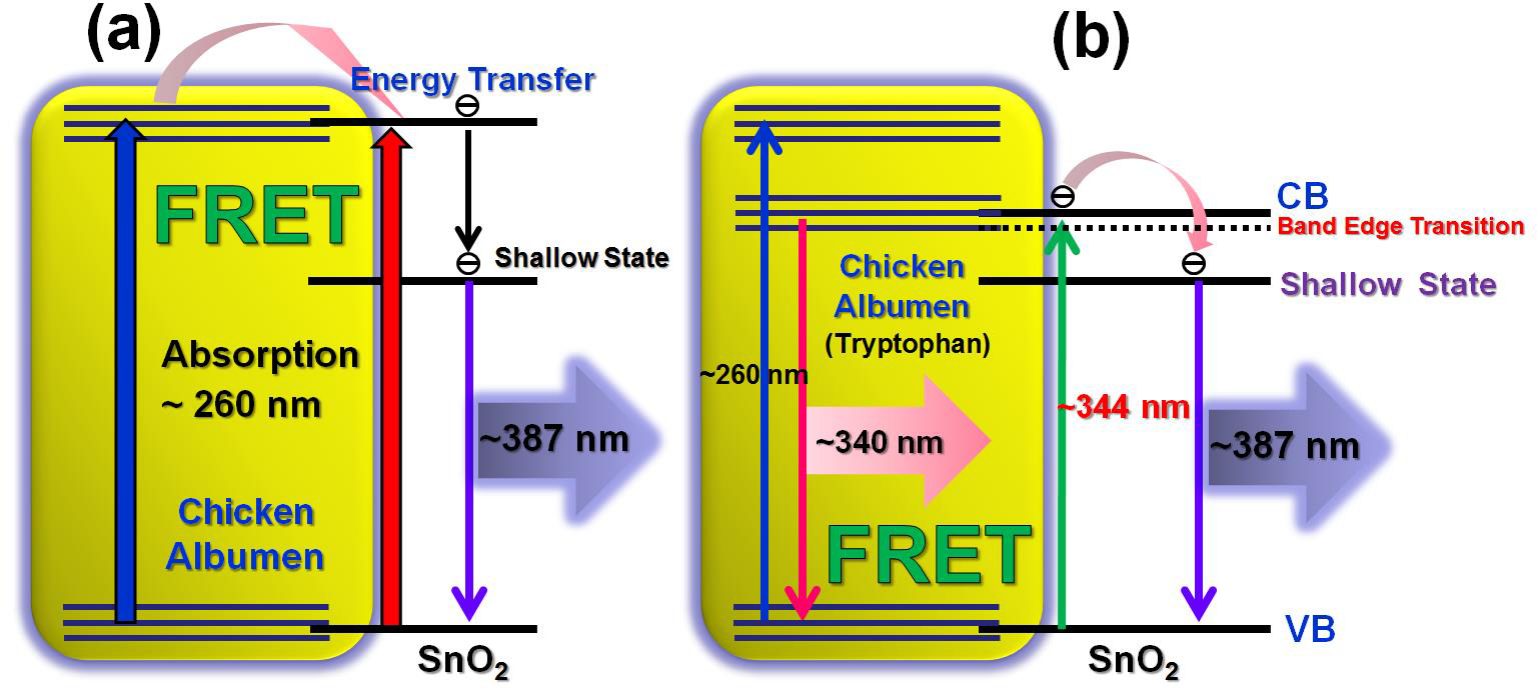
(a), (b) Illustration for the physical picture of fluorescence resonance energy transfer process existing between albumen protein and SnO_2_ nanowires.

In order to interpret the enhancement of 387 nm UV emission, the fluorescence resonance energy transfer (FRET) is believed to be the most possible underlying mechanism. For the occurrence of FRET, donor should be located in the close proximity of acceptor. Meanwhile, a good overlap is required between the optical transition bands of donor and acceptor. In our study, the donor and acceptor are albumen protein and SnO_2_ NWs, respectively. According to the previous reports^15^,^19^,^20^, the absorption spectra of albumen and SnO_2_ exhibit a good overlap ranging from 250 nm to 300 nm. It is well documented that the strong optical absorption in albumen is at around 3.65 eV, while that of SnO_2_ occurs at 3.6 eV. Therefore, the newly designed albumen/SnO_2_ NWs composite is well feasible for the proposed FRET mechanism, in which the energy can be efficiently transferred from albumen protein to SnO_2_ NWs. Figure 5 illustrates the band alignment diagram, which provides a clear physical picture to describe the FRET process between chicken albumen and SnO_2_ NWs via two dominant steps. First, in Fig. 5(a), due to the good overlap between the absorption bands of albumen and SnO_2_, the energy absorbed by the albumen protein can be easily transferred to SnO_2_ NWs through the resonant FRET effect. Second, the 340 nm UV transition arising from Tryptophan in albumen also shows an excellent overlap with the bandedge transition of 344 nm in SnO_2_ NWs thus supports the origin of FRET as well. However, limited by the direct-forbidden nature, the bandgap emission with the photon energy of 3.6 eV is prohibited. Therefore, the excited carriers will transfer to the nearest shallow-trapped state and result in the UV fluorescence with the wavelength of 387 nm (3.2 eV). Based on the above proposed mechanisms, the PL spectra shown in Fig. 4 can now be well interpreted.

**Figure 6:**
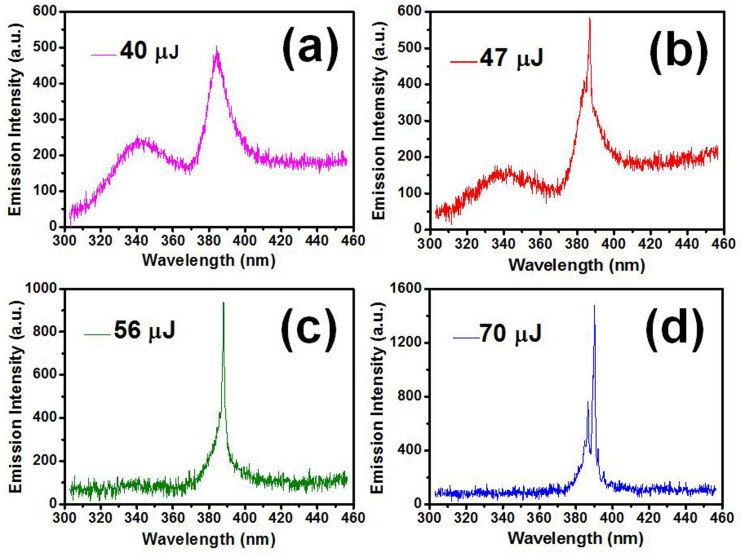
Laser actions of albumen-coated SnO_2_ nanowires under different excitation energy.

**Figure 7:**
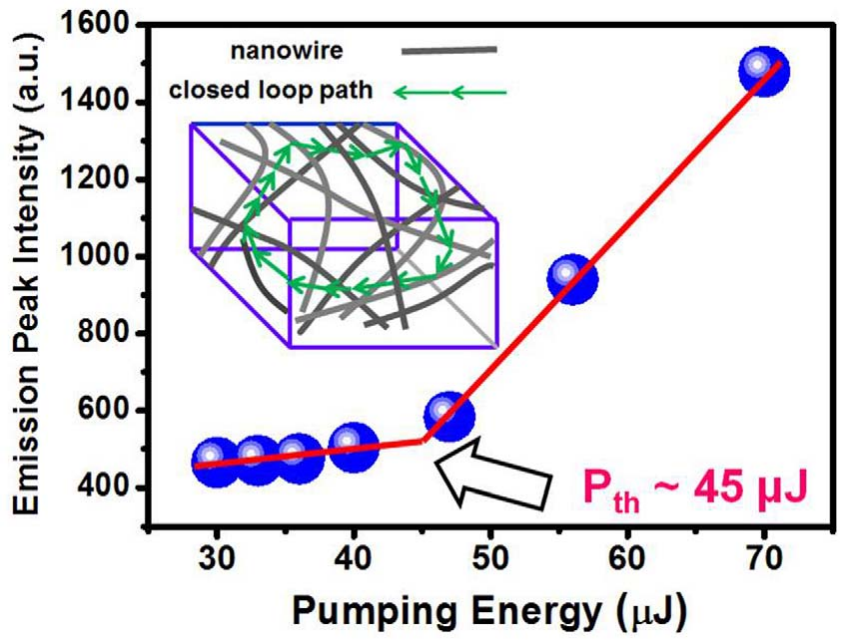
Emission peak intensity of albumen-coated SnO_2_ nanowires versus pumping energy. The inset illustrates the closed-loop path as a cavity for random laser.

Lasing occurs in a cavity by stimulated emission, which provides optical amplification^1^. Herein, the coherent optical transition within the bandedge of gain medium is important. For the occurrence of deep-trapped recombination in SnO_2_ NWs, the long decay time makes it not capable to block the fast coherent radiation of bond excitons. On the contrary, the excited carriers are much more efficient to bind and populate into the shallow trapped states, and emit photons with a fast decay rate^14^. Hence, the laser action amplified by coherent stimulated emission can be expected in the albumen/SnO_2_ NWs system at the wavelength of 387 nm rather than the visible range. To confirm our expectation, the albumen/SnO_2_ NWs composite were optically pumped by the Q-switched Nd: YAG laser (266 nm, 3–5 ns pulse, 10 Hz) for the lasing study. Series of fluorescence spectrum under different excitation energy were performed as shown in Figs. 6(a)–(d). At a low pumping power such as 40 μJ, the PL spectrum is broad and featureless (Fig. 6(a)). However, when the pumping energy exceeds a specific threshold of 45 μJ (Fig. 7), a sharp lasing peak at around 387 nm with the line width (FWHM) less than 1.3 nm starts to emerge. The rapid increase of fluorescence intensity indicates that the stimulated emission occurs in the hybrid system. As the pumping energy goes higher, the more output lasing modes appear around the wavelength of 387 nm. It is worth noting that the albumen emission located around 340 nm shows a reduction as the lasing peaks and intensity grow. As additional evidence, the reduction further supports the strong energy transfer from albumen protein to SnO_2_ NWs via the proposed resonant FRET effect.

## Discussion

For the occurrence of laser action in our study, Fabry-Perot (FP) and Whispering Gallery Mode (WGM) types will not be considered as main mechanisms, since the former requires highly oriented vertical nanowire array providing flat facets in both ends as an optical cavity, while the later needs spherical or hexagonal shape cavity as a confinement of light^21^,^22^. It is believed that the dominant mechanism can be interpreted by the random lasers (RLs). RL consists of a randomly distributed structures dispersed into an optical gain medium, in which the closed-loop path (optical cavity) is merely provided by multiple scattering process of light^23^. Therefore, the randomly assemble tetragonal SnO_2_ NWs are well feasible for the occurrence of RL. By using the information of the wavelength difference (Δλ) derived from the two nearest lasing peaks, the scattering mean free path (L) of a light in the composite system can be calculated by L = λ^2^/2nΔλ^24^, where λ is the resonant wavelength (~387 nm), n is the refractive index (~2), L is the resonant cavity length, and the approximate value is about 23.4 μm. Besides, the varied lasing modes and intensity under different pumping further confirm the RL existence in the albumen/SnO_2_ NWs composite, since the random cavity made by closed-loop path changes every moment. It should be noted that RL derived from the albumen/SnO_2_ NWs composite contains only few peaks compared with the early reports^4^. This may be explained by the reduction of scattering strength since the refractive index of albumen (n ~ 1.35) is less than that of the SnO_2_ NWs (n ~ 2.0)^25^, as well as by the narrow spectrum due to the shallow trapped state^14^. The advantage of mode reduction leads to the possibility of mode controlling and towards mode locking for the future RL applications. The emission peak intensity as a function of pumping energy is shown in Fig. 7, from which the threshold (P_th_) of about 45 μJ can be derived. The inset of Fig. 7 illustrates the closed-loop path inside nanowires. It is intriguing to further realize why the shallow-trapped state caused by impurities can produce the strong stimulated emission. In 1962, Rushba *et al*. first pointed out that the weakly bond exciton of impurities or defects can generate a giant oscillator strength, which is many orders larger than that of the free exciton^26^. The wave function generated from the weakly bond exciton basically involves a whole region around defects, thus leads to the coherent oscillations of electrons^14^,^26^. On the shallow-trapped state, there are many connected bond excitons^26^, it is therefore believed that the stimulated emission can be well generated. This theoretical work provides an excellent foundation for the understanding of our observed behavior that the energy harvesting from FRET process can be efficiently turned into the UV emission arising from shallow defects in SnO_2_ NWs.

In conclusion, we have demonstrated that a giant enhancement of 387 nm UV emission can be easily achieved from SnO_2_ NWs by the assistance of UV-emitting (340 nm) chicken albumen protein at room temperature. The underlying mechanism is interpreted by the fluorescence resonance energy transfer (FRET), in which the energy can easily transfer from albumen protein (donor) to SnO_2_ NWs (acceptor). Laser actions can be further derived from the albumen/SnO_2_ NWs composite as well. Via a careful characterization, the laser action is believed to be the RL type lasing and is generated by the stimulated emission arising from the shallow-trapped state of SnO_2_ NWs. The efficient transfer of energy harvesting from FRET process into UV emission is supported by theoretical work of extremely high oscillator strength of shallow defects in SnO_2_ semiconductors. It is believed that our work shown here not only enables to open up the possibility of utilizing various biomolecule options for improving wide fields of light emitters, but also pave a new avenue towards future green bio-inspired optoelectronic devices.

## Methods

### Sample fabrication

The n-type SnO_2_ nanowire arrays were grown on silicon (Si) substrates via vapor -liquid-solid (VLS) method. Prior to the growth, the single crystal Si substrates (0.5 cm × 0.5 cm) were ultrasonically cleaned for 10 min in acetone, ethanol and deionized (DI) water to remove any absorbed contaminant. Next, Au thin film with a thickness of 10 nm was deposited on Si substrates by using a sputtering system (JFC-1600, JEOL). A high purity (99.99%) Sn metallic powder (1.5 g) was placed on a ceramic boat and the Au-coated Si substrate is nearby the powder. The boat was then loaded to the center of a horizontal tube furnace under the Argon gas with a flow rate of 200 sccm (sccm denotes cubic centimeter per minute at STP). After the above steps, the furnace temperature was elevated to 1000°C rapidly at a rate of 100°C min^−1^. The sample was kept annealing for 10 min at 1000°C. During the process, the vaporized Sn was reacted with oxygen and resulted in SnO_2_ which blown onto the Au layer. As SnO_2_ dissolved into Au, the nucleation occurred when the alloy of SnO_2_-Au droplet reached supersaturation. After the furnace cooled down to room temperature, the as-grown white color SnO_2_ NWs were obtained on Si substrate. Albumen liquid was obtained from chicken eggs purchased from a widely seen convenience store. After separating the egg yolk, the rest albumen liquid was directly used without any further purification or post baking. To fabricate our albumen-coated SnO_2_ NWs composite, the extracted albumen was spin-coated (5000 rpm) onto the as-grown SnO_2_ NWs for 60 sec.

### Morphology characterization and optical measurements

The morphologies of SnO_2_ NWs and surface roughness of albumen were characterized by scanning electron microscope (SEM; JEOL JSM6500) and atomic force microscopy (AFM; Nanosurf Easyscan 2). The existence of SnO_2_ NWs were confirmed by the Raman scattering measurement (Jobin Yvon T64000), X-ray diffraction (XRD; Panalytical X'pert PRO), and PL spectra were carried out by the excitation of 266 nm laser. The transmittance spectra of albumen with different spin-coating speed were detected with a spectrophotometer (Jobin-Yvon H10). To study the lasing behavior, the albumen-coated SnO_2_ NWs were optically excited by a Q-switched 4^th^ harmonic Nd: YAG laser (266 nm, 3–5 ns pulse, 10 Hz) and measured with a Jobin Yvon iHR550 imaging spectrometer system. The laser beam was focused to a diameter of about 300 μm. All of the luminescence experiments were performed at room temperature.

## Author Contributions

C.S.W., T.Y.L. and Y.F.C. designed the experiment. C.S.L., T.M.S. and Y.C.C. reviewed the manuscript. C.S.W. performed the data measurements and wrote the manuscript. All authors contributed to the analysis and commend of the manuscript.

## References

[b1] Nizamoglu S., Gather M. C. & Yun S. H. All-biomaterial laser using vitamin and biopolymers. Adv. Mater. 25, 5988 (2013). 10.1002/adma201300818 24425626

[b2] Johnson J. C. *et al.* Single gallium nitride nanowire lasers. Nat. Mater. 1, 106–110 (2002). 12618824 10.1038/nmat728

[b3] Vanmaekelbergh D. & Vugt L. K. ZnO nanowire lasers. Nanoscale 3, 2783–2800 (2011). 21552596 10.1039/c1nr00013f

[b4] Yang H. Y. *et al.* Ultraviolet coherent random lasing in randomly assembled SnO_2_ nanowires. Appl. Phys. Lett. 94, 241121 (2009).

[b5] Heo J., Jahangir S., Xiao B. & Bhattacharya P. Room-temperature polariton lasing from GaN nanowire array clad by dielectric microcavity. Nano. Lett. 13, 2376–2380 (2013). 23634649 10.1021/nl400060j

[b6] Saxena D. *et al.* Optically pumped room-temperature GaAs nanowire lasers. Nat. Photonic. 7, 963–968 (2013).

[b7] Li J. *et al.* Wavelength tunable CdSe nanowire lasers based on the absorption-emission-absorption process. Adv. Mater. 25, 833–837 (2013). 23135956 10.1002/adma.201203692

[b8] Yeh P. H., Li Z. & Wang Z. L. Schottky-gated probe-free ZnO nanowire biosensor. Adv. Mater. 21, 4975–4978 (2009). 25376437 10.1002/adma.200902172

[b9] Motayed A. *et al.* Diameter dependent transport properties of gallium nitride nanowire field effect transistors. Appl. Phys. Lett. 90, 043104 (2007).

[b10] Wan Q. *et al.* Fabrication and ethanol sensing characteristics of ZnO nanowire gas sensor. Appl. Phys. Lett. 84, 3654 (2004).

[b11] Krogstrup P. *et al.* Single nanowire solar cells beyond the Shockley-queisser limit. Nat. Photonic. 7, 306–310 (2013).

[b12] Li Y. *et al.* Realizing a SnO_2_-based ultraviolet light-emitting diode via breaking the dipole-forbidden rule. Npg. Asia. Mater. 4, e30 (2012).

[b13] Zhou W. *et al.* Bound exciton and optical properties of SnO_2_ one-dimensional nanostructures. J. Phys. Chem. C 113, 1719–1726 (2009).

[b14] Liu R. B. *et al.* Stimulated emission from trapped excitons in SnO_2_ nanowires. Physica E 39, 223–229 (2007).

[b15] Chen I. T., Chang P. H., Chang Y. C. & Guo T. F. Lighting up the ultraviolet fluorescence from chicken albumen through plasmon resonance energy transfer of gold nanoparticles. Sci. Rep. 3, 1505 (2013). 23514900 10.1038/srep01505 PMC3604704

[b16] Chang J. W. *et al.* Chicken albumen dielectrics in organic field-effect transistors. Adv. Mater. 23, 4077–4081 (2011). 21809399 10.1002/adma.201102124

[b17] Cerdán L. *et al.* FRET-assisted laser emission in colloidal suspensions of dye-doped latex nanoparticles. Nat. Photonic. 6, 621–626 (2012).

[b18] Berney C. & Danuser G. FRET or no FRET: A quantitative comparison. Biophys. J. 84, 3992–4010 (2003). 12770904 10.1016/S0006-3495(03)75126-1 PMC1302980

[b19] Sinha A. K. *et al.* Tin oxide with a p-n heterojunction ensures both UV and visible light photocatalytic activity. RSC. Adv. 4, 208–211 (2014).

[b20] Wang H. *et al.* Photochemical growth of nanoporous SnO_2_ at the air-water interface and its high photocatalytic activity. J. Mater. Chem. 20, 5641–5645 (2010).

[b21] Huang M. H. *et al.* Room-temperature ultraviolet nanowire nanolasers. Science 292, 1897–1899 (2001). 11397941 10.1126/science.1060367

[b22] Chen R., Lin B., Sun X. W. & Sun H. D. Room temperature excitonic whispering gallery mode lasing from high-quality hexagonal ZnO microdisks. Adv. Mater. 23, 2199–2204 (2011). 21462376 10.1002/adma.201100423

[b23] Cao H. *et al.* Random laser action in semiconductor powder. Phys. Rev. Lett. 82, 2278–2281 (1999).

[b24] Wang C. S., Chang T. Y., Lin T. Y. & Chen Y. F. Biologically inspired flexible quasi-single-mode random laser: An integration of *Pieris canidia* butterfly wing and semiconductors. Sci. Rep. 4, 6736 (2014). 25338507 10.1038/srep06736 PMC4206842

[b25] Yang H. Y., Yu S. F., Li G. P. & Wu T. Random lasing action of randomly assembled ZnO nanowires with MgO coating. Opt. Express 18, 13647–13654 (2010). 20588498 10.1364/OE.18.013647

[b26] Rashba É. I. & Gurgenishvili G. É. Edge absorption theory in semiconductors. Sov. Phys. Solid State 4, 759–760 (1962).

